# The overall effects of virtual reality interventions on ICU patients: A systematic review and meta-analysis

**DOI:** 10.1097/MD.0000000000051180

**Published:** 2026-10-23

**Authors:** Xihui Sun, Vimala Ramoo, Jiangping Ma, Nor’azim Mohd Yunos

**Affiliations:** a Faculty of Nursing, Universiti Malaya, Kuala Lumpur, Malaysia; b School of Nursing, Faculty of Health Science, Nursing and Education, MAHSA University, Selangor, Malaysia; c Department of Anesthesiology, Faculty of Medicine, Universiti Malaya, Kuala Lumpur, Malaysia.

**Keywords:** adult patients, intensive care units, mental health, meta-analysis, pain management, sleep quality, virtual reality

## Abstract

**Background:**

Adult intensive care unit (ICU) patients frequently experience psychological distress, pain, sleep disruption, and motor impairments. Virtual reality (VR) has emerged as a promising nonpharmacological adjunct to improve these outcomes, yet existing evidence remains fragmented and inconsistent. This systematic review and meta-analysis synthesized recent clinical trials to evaluate the effectiveness of VR on mental health, pain, sleep, functional outcomes, and delirium in adult ICU patients.

**Methods:**

This review followed Preferred Reporting Items for Systematic Reviews and Meta-Analyses (2020) guidance and was registered in International Prospective Register of Systematic Reviews (CRD42023482529). Eight databases were searched for randomized controlled trials published between January 2000 and September 2025. Eligible studies included adult ICU patients receiving immersive or non-immersive VR interventions. Primary outcomes were anxiety, depression, and post-traumatic stress disorder (PTSD); secondary outcomes included pain, sleep quality, lower-limb function, and bedside balance. Risk of bias was assessed using the Cochrane RoB tool, and certainty of evidence was graded using Grading of Recommendations Assessment, Development and Evaluation.

**Results:**

Twenty randomized controlled trials were identified, involving a total of 1551 patients. This meta-analysis showed that VR intervention helped improve anxiety (standardized mean difference [SMD] = −0.67, 95% confidence interval [CI: −1.00 to −0.35], *P* < .0001), depression (SMD = −0.46, 95% CI [−0.72 to −0.21], *P* = .0004), PTSD [mean difference = −5.48, 95% CI (−9.36 to −1.59), *P* = .006], lower-limb function score [mean difference = 2.83, 95% CI (1.99 to 3.67), *P* < .001] and sleep quality (SMD = 0.70, 95% CI [0.47 to 0.92], *P* < .0001), but had no significant effect on pain score (SMD = −0.57, 95% CI [−1.49 to 0.36], *P* = .23) and bedside balance score (SMD = 0.61, 95% CI [−0.44 to 1.66], *P* = .25). Most studies were assessed as having varying degrees of risk of bias. All evidence was rated from very low to moderate.

**Conclusion:**

VR interventions may confer meaningful benefits for anxiety, depression, PTSD, and sleep quality in adult ICU patients, and may improve lower-limb function in selected populations. However, evidence for analgesic effects and bedside balance remains inconclusive. Standardized VR protocols, objective outcome assessment, and adequately powered multicenter trials are needed to clarify the clinical value of VR in ICU care.

## 1. Introduction

Intensive care unit (ICU) survivors frequently experience post-intensive care syndrome, characterized by persistent disturbances in mental health, cognition, pain, sleep quality, and motor function.^[1]^ These sequelae arise from complex stressors, including restrictive visitation policies,^[2]^ constant alarm noise,^[3]^ prognosis-related uncertainty,^[4]^ and discomfort from invasive procedures.^[5]^ Significant psychological distress (e.g., anxiety and depression) and motor impairment are common among ICU patients. Emotional distress is highly prevalent, with anxiety and depression affecting 29% and 34% of ICU patients respectively,^[6,7]^ and even higher rates reported in China.^[8,9]^ Pain is also a prevalent issue among ICU patients, often stemming from tissue damage, invasive procedures, and psychological factors like anxiety and depression.^[10,11]^ Pain, driven by both physical injury and psychological factors, affects 50% of ICU patients at rest and up to 80% during routine care.^[12]^ Prolonged analgesic or sedative use, commonly employed in ICUs, further contributes to dependence, impaired mobility, and disrupted sleep.^[13]^

Motor dysfunction – including impaired strength, mobility, balance, and endurance –is another hallmark of critical illness.^[14,15]^ ICU-acquired weakness affects 25% to 100% of patients, particularly those mechanically ventilated or immobilized.^[16]^ Sleep disturbances are similarly widespread; 68% of Chinese ICU patients experience severe disruptions, with consequences for mental status, cognition, and long-term recovery.^[17]^ The cumulative symptoms of post-intensive care syndrome are associated with poorer quality of life,^[18]^ underscoring the urgent need for adjunctive nonpharmacological interventions to complement conventional ICU care.^[19]^

Although psychosocial interventions such as music therapy, cognitive behavioral therapy, early mobilization, sleep-promotion strategies, and ICU diaries have been explored,^[20–23]^ their real-world feasibility and effectiveness remain inconsistent. Music therapy requires environmental control and patient stability.^[24]^ Early mobilization demands substantial staffing and patient readiness,^[25]^ but faces limitations related to standardization, privacy, and staff workload.^[26]^ These limitations highlight the need for interventions that are safe, feasible, minimally burdensome, and able to address the multidimensional symptoms of ICU patients.

Virtual reality (VR) technologies offer a compelling alternative by providing immersive multisensory environments designed to modulate attention, reduce stress, and enhance emotional regulation.^[27,28]^ Immersive VR delivered through head-mounted displays generates high presence and deep sensory engagement, while semi-immersive or non-immersive VR offers more moderate stimulation via tablets or 2D screens.^[29]^ Evidence suggests immersive and interactive VR experiences – including natural landscapes or game-based tasks – can reduce distress, distract from pain, improve cognitive engagement, and potentially promote motor participation.^[30–33]^ Early VR studies in ICUs focused primarily on feasibility and safety, generally reporting minimal adverse effects and favorable patient acceptance.^[34–36]^ Subsequent randomized controlled trials (RCTs) have demonstrated reductions in symptoms of anxiety, depression, and post‑traumatic stress disorder (PTSD).^[37–41]^ However, effects on pain, sleep quality, motor function, and delirium remain inconsistent across studies.^[42–44]^ These discrepancies may reflect substantial heterogeneity in VR modalities, session duration, patient demographics, disease severity, and outcome measures. Moreover, previous reviews have often focused on isolated symptoms (e.g., anxiety or stress),^[43,45]^ included a limited number of trials, or failed to assess emerging outcomes such as sleep or mobility. Crucially, as new relevant evidence emerges, the effects of VR on ICU patients’ limb function, pain and sleep quality require reevaluation.^[30,38,46,47]^ Furthermore, prior syntheses neither rigorously explored heterogeneity using sensitivity analyses nor employed an evidence‑grading framework to evaluate evidence certainty.

Given these limitations, a robust synthesis of contemporary trials is needed to clarify VR’s overall therapeutic value for ICU patients. Therefore, this systematic review and meta-analysis aimed to evaluate the effects of VR on mental health, pain, sleep outcomes, and physical function among adult ICU patients by synthesizing evidence from recent RCTs.

### 1.1. Aims

To systematically evaluate the effects of VR technology on the mental health, pain levels, sleep quality, and motor function of ICU patients, with a focus on content variability and delivery mechanisms.

## 2. Materials and methods

### 2.1. Inclusion and exclusion criteria

#### 2.1.1. Inclusion criteria

The items for inclusion eligibility were as follows: articles published in Chinese or English; participants were ICU adults aged 18 to 70; RCTs only; control group receiving standard care or controlled VR interventions, such as basic care (such as body cleaning and warming measures), conventional rehabilitation (active or passive limb exercises), and usual psychological care (family visits and verbal reassurance); patients in the experimental group received VR interventions that included natural scene simulations (such as beaches, lakes or coral reefs), somatosensory games (such as meditation, driving, or rehabilitation training), cognitive exercises, and relaxing music or video playbacks. These interventions could be highly immersive scenes (played through VR glasses) or moderately non-immersive scenes (via widescreen VR technology). Finally, the outcome measures included any items related to anxiety, depression, PTSD, pain, motor function, delirium rate, and sleep quality.

#### 2.1.2. Exclusion criteria

The exclusion criteria were as follows: patients from pediatric ICUs or general hospital wards; review articles or case reports; clinical trials without a control group; duplicate publications; studies with unavailable data; and outcome measures inconsistent with the prespecified meta-analytic analysis plan.

### 2.2. Search strategy

Two researchers independently performed a comprehensive search through the search strategy designed by librarians. The following terms and combinations were used in searches across all databases: “virtual reality” or “VR devices” or “virtual reality technology” or “electronic relaxation devices,” and “ICU patients” or “intensive care patients” or “critically ill patients,” and “pain” or “anxiety” or “post-traumatic stress disorder” or “post-intensive care syndrome” or mental health’ or “motor function” or “sleep quality,” and “randomized controlled trials” or “clinical randomized trials.” For details about search strategy, please refer to File S1, Supplemental Digital Content 1. The relevant journals and bibliographies were manually searched. In case of any disputes regarding the final inclusion of literature, a third evidence-based researcher would be consulted to reach a final agreement. Searches were performed in 2025 across 8 electronic databases for studies published from January 2000 to September 2025, including the Cochrane Library, Web of Science, PubMed, Cumulative Index to Nursing and Allied Health Literature, China National Knowledge Infrastructure, Embase, ScienceDirect, and Wiley Online Library. Grey literature was additionally retrieved via Google Scholar and ProQuest Dissertation & Theses Global.

### 2.3. Design

This systematic review was registered with International Prospective Register of Systematic Reviews (CRD42023482529) and conducted in accordance with the Preferred Reporting Items for Systematic Reviews and Meta-Analyses guidelines.^[48]^ Two modifications were made to the registered protocol. First, the term “psychological symptoms” was revised to “mental health” to provide clearer and more consistent terminology for the psychological outcomes included in the review. Second, lower-limb function and bedside balance were analyzed as separate functional outcomes in the meta-analysis due to sufficient synthesizable data. These revisions kept the study population, intervention, comparator, main review objectives, and eligibility criteria unchanged.

### 2.4. Literature screening and data extraction

Two independent researchers jointly developed data-extraction forms and reached consensus on the data-extraction process and literature quality assessment. Information extracted from included studies covered: first author’s name, publication year, country, ICU setting, study design, sample size, intervention protocol, control conditions, outcome measures, adverse events and measurement methods. The primary outcome indicators for this meta-analysis were levels of anxiety, depression and PTSD; while secondary outcomes included pain scores, lower-limb mobility, sleep quality and bedside balance.

### 2.5. Literature quality assessment

The risk of bias in each RCT article was assessed by 2 researchers using Cochrane recommended risk assessment criteria.^[49]^ The evaluation criteria include 7 elements including random method and hidden allocation. For details on the risk assessment tools used in the study, please refer to File S2, Supplemental Digital Content 2. To ensure consistency and accuracy, 2 researchers skilled in evidence-based medicine independently conducted data extraction and quality assessment. A third expert in evidence-based medicine would resolve any substantial discrepancies.

### 2.6. Clinical evidence grading

Certainty of evidence for each outcome was assessed using the Grading of Recommendations Assessment, Development and Evaluation (GRADE) approach (GRADE‑Pro v3.6.1). Evidence certainty was rated across 5 GRADE domains: risk of bias, inconsistency, imprecision, indirectness, and publication bias. Evidence certainty was categorized into 4 grades: very low, low, moderate, and high.^[50]^

### 2.7. Statistical methods

This meta‑analysis was performed using Review Manager 5.4. Heterogeneity among studies was assessed using Cochran Q test and the *I^2^* statistic. The *I^2^* statistic was used to describe the proportion of variability in effect estimates attributable to between-study heterogeneity rather than sampling error.^[51]^ Model selection considered both statistical heterogeneity and the anticipated clinical and methodological variability among the included studies. A random-effects model was applied when meaningful between-study heterogeneity was expected because of differences in patient characteristics, ICU settings, VR intervention modalities, intervention duration, frequency, or outcome measurement. A fixed-effect model was used when the included studies were sufficiently homogeneous and it was reasonable to assume a common underlying intervention effect.^[52]^ Sensitivity analyses were performed by comparing alternative statistical models where appropriate to determine the robustness of the pooled estimates. Continuous outcomes were summarized using either the mean difference (MD) or standardized mean difference (SMD), with corresponding 95% confidence intervals (CIs). MD was used when studies assessed an outcome using the same measurement instrument and scale, whereas SMD was used when conceptually similar outcomes were assessed using different measurement instruments or scales. The selection of MD or SMD was therefore based on the comparability of the outcome measurement scales and was independent of the degree of statistical heterogeneity.^[53]^

## 3. Results

### 3.1. Study screening process and search summary

EndNote X9 was used to manage retrieved citations. First, a total of 1404 studies were identified through online literature searches. After removing duplicates and applying exclusion criteria, 544 studies remained, of which 510 were excluded due to topic and design. Thirty-four studies were selected for eligibility review. Twenty RCTs were ultimately deemed suitable for inclusion in this meta-analysis and systematic review.^[30–34,37–41,46,47,54–61]^ The screening process of the study is shown in Figure 1.

**Figure 1.**
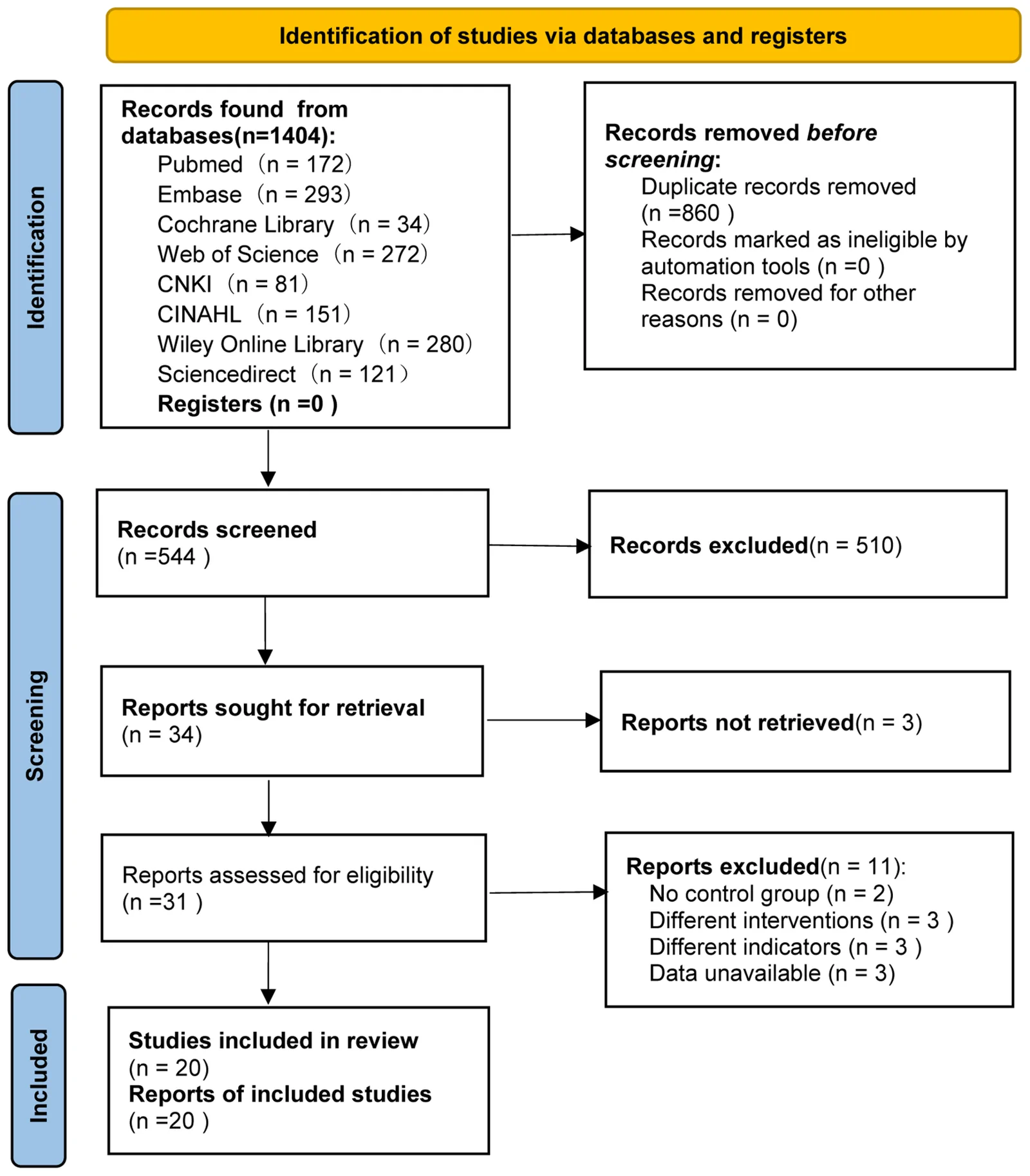
PRISMA flow diagram. Researchers screened and excluded literature based on PRISMA 2020. CINAHL =Cumulative Index to Nursing and Allied Health Literature, CNKI=China National Knowledge Infrastructure, PRISMA =Preferred Reporting Items for Systematic Reviews and Meta-Analyses.

### 3.2. Study characteristics

The sources of the included studies were diverse: 8 were from China,^[31,37,38,47,54,55,58,59]^ 3 from France^[41,57,62]^ and 2 from the Netherlands.^[34,40]^ There was also 1 study each from Belgium, Korea, Spain, Iran, Turkey, and Italy.^[32,33,39,46,56,60,61]^ Pertaining to the origins or development processes of VR interventions in the studies, 3 studies mentioned that the interventions were developed by multidisciplinary teams and underwent expert validation.^[33,34,40]^ Six studies utilized VR videos or panoramic landscapes provided by the VR devices themselves.^[41,56,57,60–62]^ One study sourced its VR content from free 360° videos available on YouTube,^[39]^ while 4 studies used rehabilitation training programs embedded in VR systems.^[31,32,58]^ Table 1 presents details of the literature.

**Table 1:** Characteristics of RCT studies (n = 20)

References/ Country	Sample E/C	ICU type	Intervention program		Adverse events of VR	Measuring tools	Outcome indicator
			Experimental group	Control group			
			Content, frequency and duration of VR				
Kim and Kang^[46]^ Korea	47:49	SICU	*Immersive VR meditation*: Four 20-min modules via HM-VR over 2 nights.	Usual care	NR	VSHSS	⑤
Yan et al^[37]^ China	39:41	CCU	*ICU-VR preop education*: 10-min single VR session on ICU environment and postoperative self-care.	Usual care	NR	SASS, RCSQ	①⑤
Li et al^[38]^ China	70:78	CCU	*Immersive VR cognitive intervention*: 30 min/d for 7 d, self-selected hypnosis/mindfulness/music/relaxation modules.	Usual care	Dizziness Headache	HAM-A, PSQI	①⑤
Dalir et al^[30]^ Iran	35:35	MICU	*Immersive VR*: self-selected nature video (~10 min) around CTR; pain measured pre-, post-, and 15 min post-procedure.	Usual care	NR	VAS	④
Zhu et al^[47]^ China	30:30	MICU	*Immersive VR*: 2-wk early recovery via interactive VR games.	Usual care	NR	BBS	⑦
Han et al^[54]^ Taiwan, China	35:35	MICU	*Immersive VR*: “Grasp Fruit” game started ≤ 48 h post-admission, 20 min twice daily for 3 d.	Usual care	NR	DASS, RCSQ, CPOT	①④⑤
Castelli et al^[32]^ Italy	12:12	NICU	*Non-immersive VR bed-cycling*: 3 wks, 5 d/wk; 15→20→40 min per session (weeks 1–3).	Usual care	NR	MI-LL	⑥⑦
Xia et al^[31]^ China	37:31	MICU	*Immersive VR exercise*: headset-based park/gym activities (e.g., bird-catching, tennis, and yoga); dose not reported. Dose: as tolerated from ICU day 1 to ICU discharge.	Usual care	NR	MRC	⑧
Yesilot et al^[39]^ Türkiye	55:55	SICU	*Immersive VR relaxation*: one 30 ± 5 min panoramic nature-video session on postoperative day 1.	Usual care	NR	NPRS; FAS	①④
Vlake et al^[40]^ Netherlands	45:44	MICU clinic	*Immersive VR education*: single 14-min ICU orientation session via headset.	Usual care	NR	IES-R; HADS	①②③
Rousseaux et al^[56]^ Belgium	15:22	CCU	*Immersive VR relaxation*: 360° nature, 20 min/session, twice daily for 3 d (pre/post-op).	Usual care	Fatigue	VAS	①④
Merliot-Gailhoustet et al^[57]^ France	53:56	MICU	*Immersive VR relaxation*: 2 × 15-min sessions using breathing/hypnosis nature videos (4 options).	Regular relaxation methods	Agitation and vertigo	ANI. NPRS	①④
Ouyang ^[55]^ China	25:25	MICU	*Immersive relaxation VR training*: View 360° panoramic videos of natural elements through the glasses, a total of 10 times.	Usual care		HADS-A, RCSQ	①⑤
Vlake et al^[34]^ Netherlands	23:19	MICU	*Immersive VR education*: post-ICU (≤4 d) VR on care processes, self-paced ≥ 1/d for ≥ 10 min; total duration unclear.	VR of natural view	NR	IES-R. BDI	②③
Zeng^[58]^ China	38:38	MICU	*Non-immersive VR rehab*: 2–3 games/d (20–30 min), 5 d/wk for 8 wks.	Usual care	NR	FMA. BBS	⑥⑦
Mo et al^[59]^ China	46:46	MICU	*Non-immersive VR cognitive training*: 30–45 min/session, daily, 5×/wk for 3 mo (plus usual care).	Routine mobilization	NR	FMA, BBS,	⑥⑦
Navarra-Ventura et al^[33]^ Spain	21:21	MICU	*Non-immersive cognitive training*: Non-immersive VR cognitive training: 15–20 min each morning from ICU day 2 to discharge (≤28 d).	Usual care	NR	HADS. DTS	①③
Laghlam et al^[41]^ France	90:90	CCU	*Immersive VR in nature scenes*: 2 ≥ 15-min sessions (total ≥ 30 min) around surgery and drain removal (pre/post).	Analgesic care	NR	NPRS;	①④
Lee et al^[60]^ Korea	24:24	MICU	*Immersive relaxation VR training*: Night of admission: one 30-min VR meditation/music session (8 nature scenes).	Usual care	NR	PSQI	⑤
Hessabi et al^[61]^ Iran	30:30	CCU	*Immersive VR relaxation*: 15-min nature videos on nights 2–3; total course unclear.	Usual care	NR	SSTAI	①

Characteristics of the included randomized controlled trials (n = 20). Data were summarized based on the extracted information from each eligible study. *Outcome indicators*: ① Anxiety level, ② Depression level, ③ Post Traumatic Stress Disorder Level, ④ Pain score, ⑤ Sleep quality, ⑥Lower-limb function score, ⑦Bedside balance, and ⑧Muscle strength.

ANI = Analgesia/Nociception Index, BBS = Berg Balance Scale, BDI = Beck Depression Inventory, CCU = cardiovascular intensive care unit, CPOT = Critical-Care Pain Observation Tool, DASS = Depression Anxiety Stress Scales, DTS = Davidson Trauma Scale, FAS = Faces Anxiety Scale, FMA = Fugl-Meyer assessment, HADS = Hospital Anxiety and Depression Scale, HAM-A = Hamilton Anxiety Rating Scale, IES-R = Impact of Event Scale-Revised, MI-LL = Motivation and Involvement Index for Lower Limb, MICU = mixed intensive care unit, MRC = Medical Research Council, NICU = neurology intensive care unit, NPRS = Numeric Pain Rating Scale, NR = not reported, PQSI = Pittsburgh Sleep Quality Index, RCSQ = Richards–Campbell Sleep Questionnaire, RCT = randomized controlled trial, SICU = surgical intensive care unit, SSTAI = Spielberger State-Trait Anxiety Inventory, VAS = Visual Analogy Scale, VR = virtual reality, VSHSS = Verran and Snyder-Halpern Sleep Scale.

### 3.3. Quality assessment of literature

Using the assessment tools mentioned previously, 2 independent researchers conducted a thorough assessment of the included literature and reached a consensus regarding final inclusion. Of the 20 RCTs, 8 studies used computer-aided randomization.^[32,34,38–40,54,57,61]^ The remaining 4 studies used the random number table method.^[31,37,47,55,58]^ One study used a block randomization.^[30]^

Six studies specifically mentioned the use of opaque sealed envelopes to conceal allocation.^[31,33,39,46,54,57]^ Only 1 study achieved double-blinding for both participants and researchers.^[31]^ Additionally, 4 studies specified blinding of outcome assessors.^[34,39,40,57]^ The evaluation results showed that the methodological design of 12 studies was at a moderate level, with some studies exhibiting a high risk of bias in randomization and blinding. Table 2 summarizes the methodological details of the included RCTs. Figure 2 shows the overall bias in the included literature. The 2 independent researchers gave almost identical assessments of the quality of the included literature.

**Table 2:** Risk of bias assessment for RCTs.

References	Random method	Allocation concealment	Blind method		Data integrity	Selective reporting	Other bias
			For subjects and intervener	For outcome surveyor			
Kim and Kang^[46]^	Low	Low	Unclear	Unclear	Low	Low	Low
Dalir et al^[30^	Low	Unclear	High	Unclear	Low	Low	Low
Yan et al^[37]^	Low	Unclear	Unclear	Low	Low	Low	Unclear
Li et al^[38]^	Unclear	Unclear	Unclear	Low	Low	Low	Unclear
Zhu et al^[47]^	Low	Unclear	Low	Low	Low	Low	Unclear
Han et al^[54]^	Low	Low	Low	Unclear	Low	Low	Unclear
Castelli et al^[32]^	Low	Unclear	Unclear	Unclear	Low	Low	Unclear
Xia et al^[31]^	Low	Low	Low	Unclear	Low	Low	Unclear
Ouyang et al^[55]^	Low	Unclear	Unclear	Low	Low	Low	Unclear
Rousseaux et al^[56]^	Unclear	Unclear	Unclear	Unclear	Low	Low	Unclear
Yesilot et al^[39]^	Low	Low	High	Low	Low	Low	Low
Vlake et al^[40]^	Low	Unclear	High	Low	Low	Low	Low
Merliot-Gailhoustet et al^[57]^	Low	Low	Unclear	Low	Low	Low	Low
Navarra-Ventura et al^[33]^	Unclear	Low	Unclear	Unclear	Low	Low	Unclear
Mo et al^[59]^	Low	Low	Unclear	Unclear	Low	Low	Unclear
Zeng^[58]^	Low	Unclear	Unclear	Unclear	Low	Low	Unclear
Vlake et al^[34]^	Low	Low	High	Low	Low	Low	Unclear
Laghlam et al^[41]^	Low	Unclear	Unclear	Unclear	Low	Low	Low
Hessabi et al^[61]^	Unclear	Unclear	Unclear	Unclear	Low	Low	Unclear
Lee et al^[60]^	Unclear	Unclear	Unclear	Unclear	Low	Low	Unclear

A total of 20 RCTs were assessed using the Cochrane risk-of-bias tool.

RCT = randomized controlled trial.

**Figure 2.**
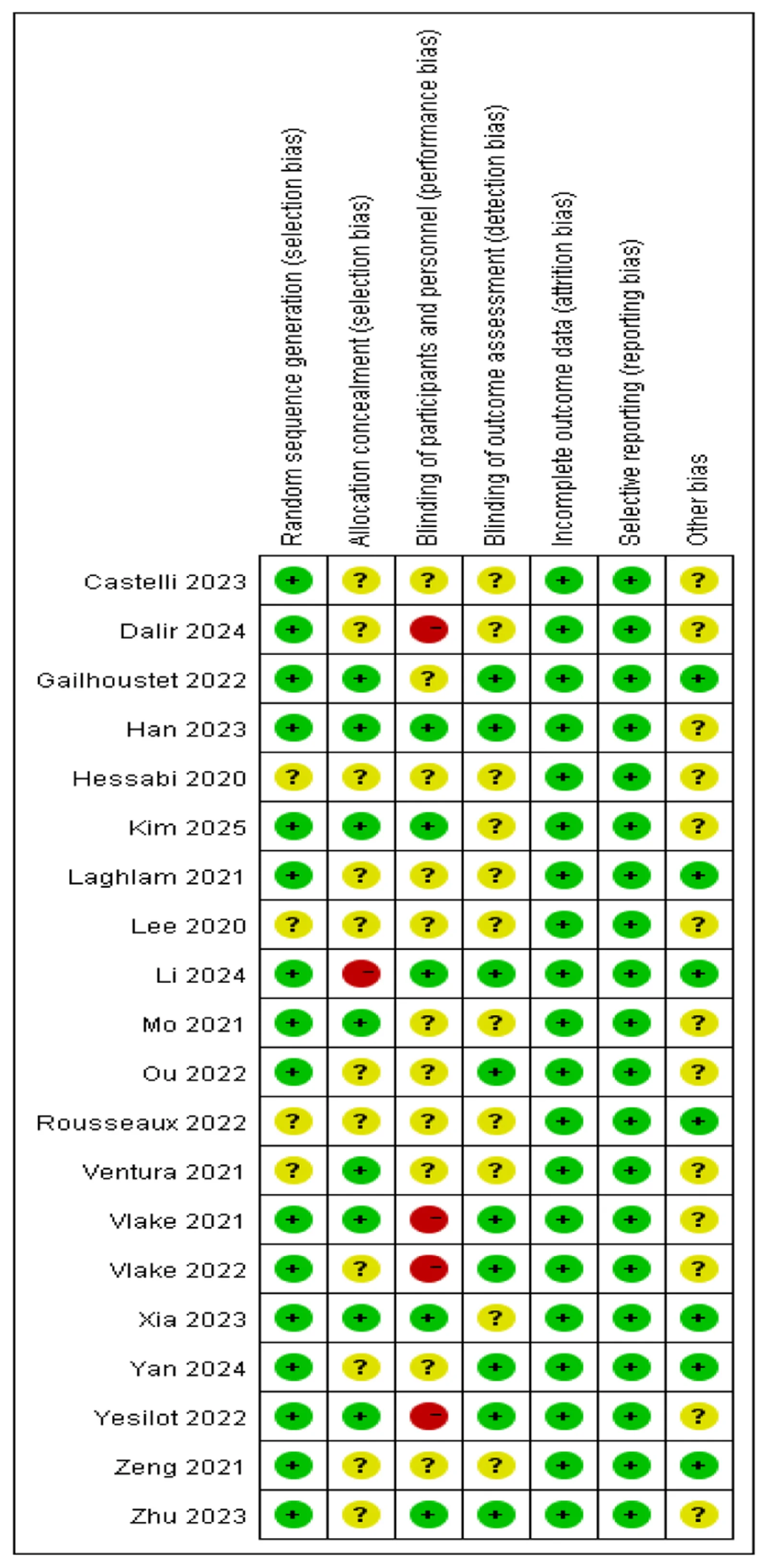
Risk of bias plot for included RCTs. Risk of bias was assessed using the Cochrane Risk of Bias tool across standard domains. RCTs = randomized controlled trials.

### 3.4. Results of the meta-analysis

#### 3.4.1. Primary outcomes

##### 3.4.1.1. Anxiety score

Ten studies involving 913 patients were included in the meta-analysis.^[33,37–41,54–57]^ Substantial heterogeneity was observed among the studies (*I^2^ *= 81%, *P* < .001). Using a random-effects model, the pooled analysis showed that VR interventions were associated with significantly lower anxiety scores compared with the control group (SMD* *= −0.67, 95% CI [−1.00 to −0.35], *P* < .0001; Fig. 3).

**Figure 3.**
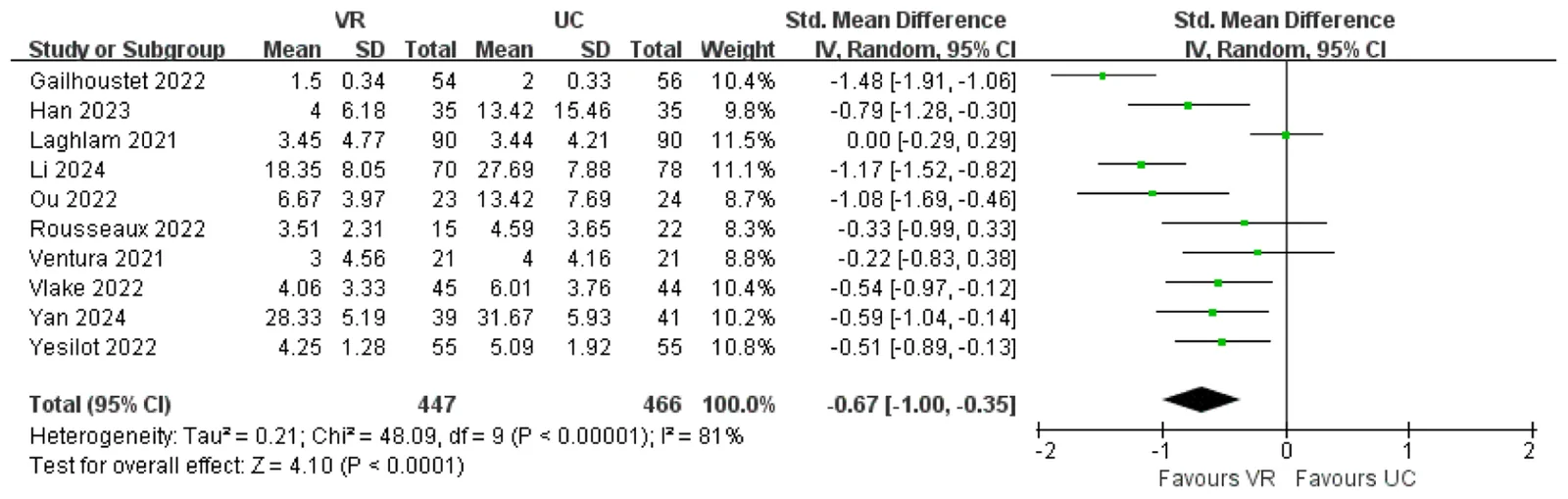
Forest plot of anxiety level. Effect sizes are presented as standardized mean differences with 95% confidence intervals. CI = confidence interval, VR = virtual reality.

##### 3.4.1.2. Depression score

Four studies with 243 patients were included in the synthesis.^[33,34,40,54]^ Low-to-moderate heterogeneity was observed among the studies (*I^2^ *= 36%). Using a fixed-effect model, the pooled analysis showed that VR interventions were associated with significantly lower depression scores compared with the control group (SMD = −0.46, 95% CI [−0.72 to −0.21], *P* = .0004; Fig. 4).

**Figure 4.**
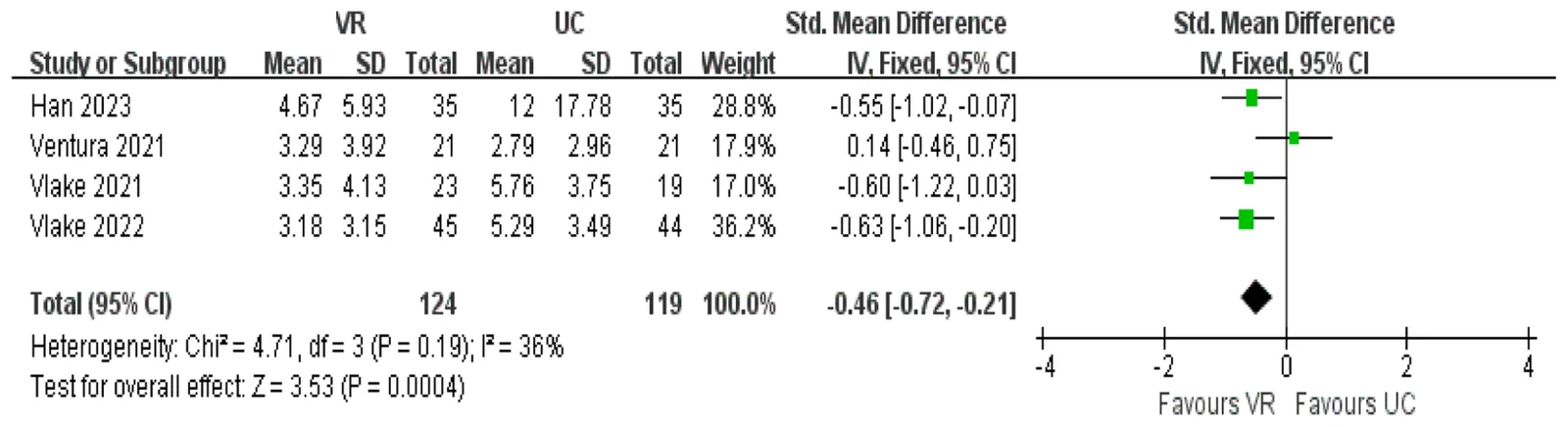
Forest plot of depression level. Effect sizes are presented as standardized mean differences with 95% confidence intervals. CI = confidence interval, VR = virtual reality.

##### 3.4.1.3. Score of PTSD

Three studies with 173 patients were included in the synthesis.^[33,34,40]^ The results of the heterogeneity test indicated minimal heterogeneity, with *I^2^* = 0% and *P* = .66. Fixed-effects model analyses showed that VR group was more effective in improving PTSD (MD* *= −5.48, 95% CI [−9.36 to −1.59], *P* = .006; see Fig. 5).

**Figure 5.**
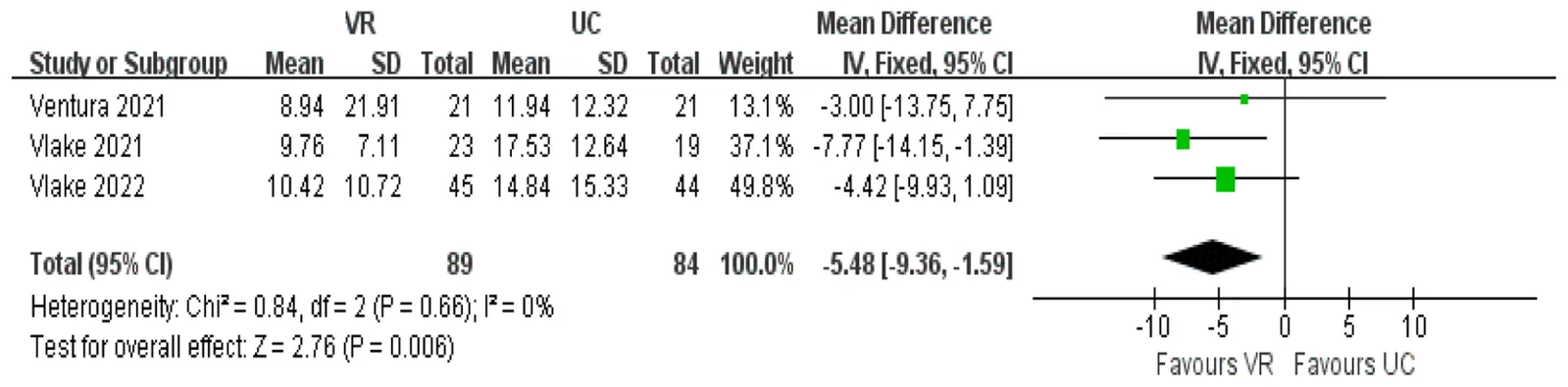
Forest plot of post-traumatic stress disorder. Effect sizes are presented as mean differences with 95% confidence intervals. CI = confidence interval, VR = virtual reality.

#### 3.4.2. Secondary outcomes

##### 3.4.2.1. Pain score

Five studies with 504 patients were included in the synthesis.^[30,39,41,56,57]^ The results of the heterogeneity test indicated marked heterogeneity, with *I^2^* = 96% and *P* < .001. The random-effects model analysis indicated that VR group showed no more significant improvement in pain relief (SMD = −0.57, 95% CI [−1.49 to 0.36], *P* = .23; see Fig. 6).

**Figure 6.**
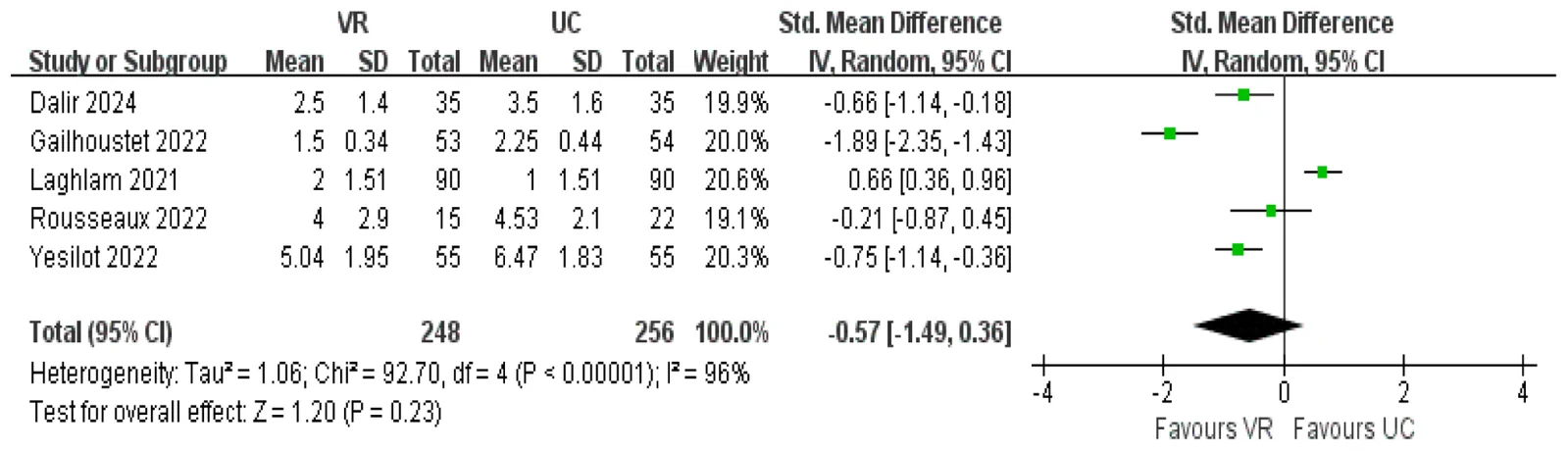
Forest plot of pain score. Effect sizes are presented as standardized mean differences with 95% confidence intervals. CI = confidence interval, VR = virtual reality.

##### 3.4.2.2. Sleep quality

Five studies with 328 patients were included in the synthesis.^[37,46,54,55,60]^ The results of the heterogeneity test indicated minimal heterogeneity, with *I^2^ = *11% and *P = *.34. Fixed-effects model analysis showed significant difference between 2 groups (SMD* *= 0.70, 95% CI [0.47 to 0.92], *P < .0001*, see Fig. 7).

**Figure 7.**
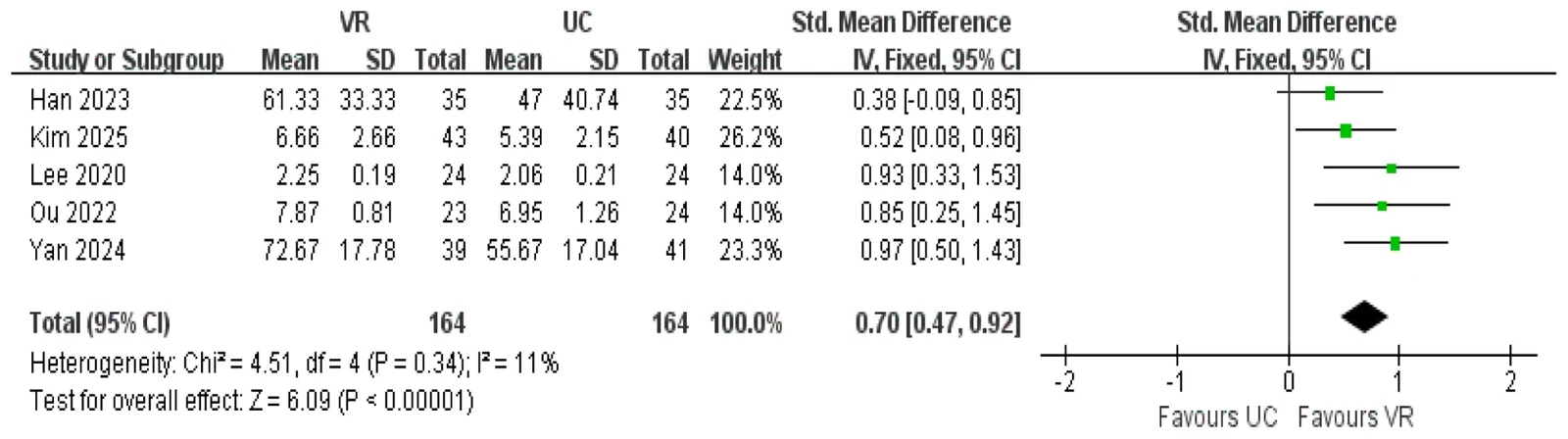
Forest plot of the sleep quality. Effect sizes are presented as standardized mean differences with 95% confidence intervals. CI = confidence interval, VR = virtual reality.

##### 3.4.2.3. Lower-limb function score

Three studies with 192 patients were included in the synthesis.^[32,58,59]^ The heterogeneity test results indicated minimal heterogeneity, with *I^2^* = 0% and *P *= .92. Fixed-effects model analyses showed that the VR group was more dominant (MD = 2.83, 95% CI [1.99 to 3.67], *P* < .0001; see Fig. 8).

**Figure 8.**
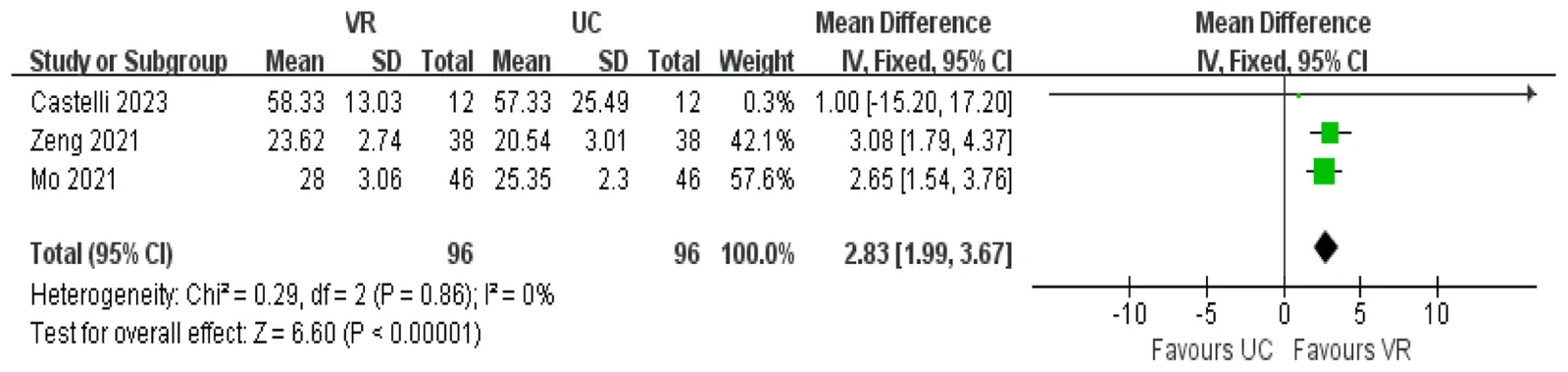
Forest plot for lower-limb function score. Effect sizes are presented as mean differences with 95% confidence intervals. CI = confidence interval, VR = virtual reality.

##### 3.4.2.4. Bedside balance score

Three studies involving 160 patients were included in the meta-analysis.^[32,47,58]^ Substantial heterogeneity was observed among the studies (*I^2^ *= 89%, *P* < .001). Using a random-effects model, the pooled analysis showed no statistically significant difference in bedside balance scores between the VR intervention and control groups (SMD = 0.61, 95% CI [−0.44 to 1.66], *P* = .25; Fig. 9). Given the substantial between-study heterogeneity, this finding should be interpreted with caution.

**Figure 9.**
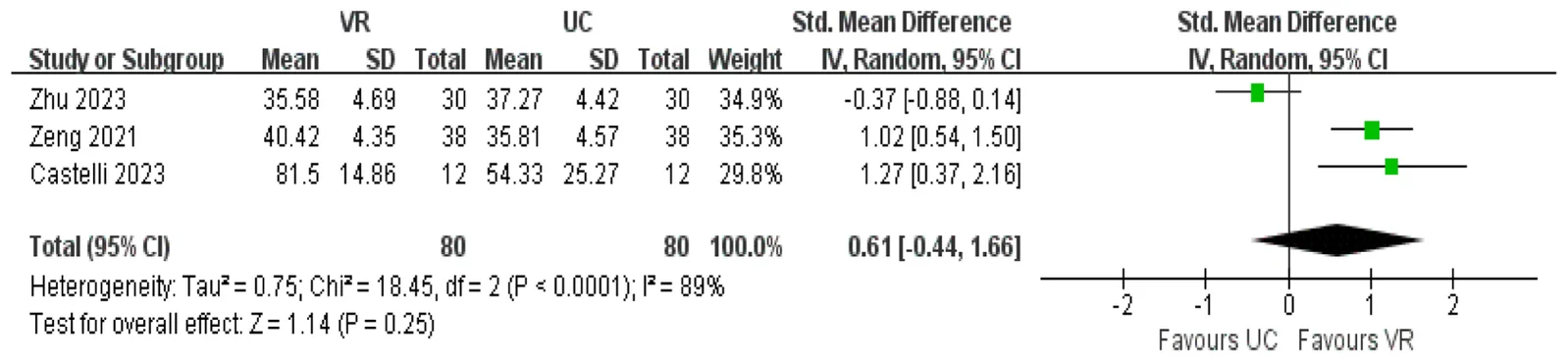
Forest plot for bedside balance. Standardized mean differences with 95% confidence intervals were presented. CI = confidence interval, VR = virtual reality.

### 3.5. Adverse events

Adverse events associated with VR interventions were reported in a limited number of studies. As shown in Table 1, Li et al reported transient dizziness and headache among participants during immersive VR sessions.^[38]^ Two additional studies reported mild agitation, fatigue and dizziness.^[56,57]^ These events were self-limiting and did not result in withdrawal from the intervention. No serious VR-related adverse events occurred across studies. Reported events were infrequent, mild, and transient, with no device interference, cardiovascular instability, or need for intervention. Safety outcomes were either unreported or indicated no harmful events.

### 3.6. Sensitivity and publication bias analysis

Sensitivity analyses were conducted by comparing the pooled estimates obtained using alternative statistical models. The direction and statistical significance of most pooled estimates remained stable. However, the findings for pain and bedside balance were sensitive to model specification, indicating that these results were less robust and should be interpreted with caution. The detailed results of the sensitivity analyses are presented in Table 3.

**Table 3:** Results of meta-analysis.

Outcome indicator	Number of studies	*I* ^2^	*P*	Meta-analysis results				Sensitivity analysis results			
				Effect mode	Effect value	95% CI	*P*	Effect mode	Effect value	95% CI	*P*
Anxiety score	10	81	<.01	Random	−0.67	(−1.00 to −0.35)	<.0001	Fixed	−0.61	(−0.75 to −0.48)	<.001
Depression score	4	36	.19	Fixed	−0.46	(−0.72 to −0.21)	.0004	Random	−0.44	(−0.77 to −0.11)	.008
Score of PTSD	3	0	.66	Fixed	−5.48	(−9.36 to −1.59)	.006	Random	−5.48	(−9.36 to −1.59)	.006
Pain score	5	96	<.01	Random	−0.57	(−1.49 to 0.36)	.23	Fixed	−0.34	(−0.53 to −0.16)	.0003
Sleep quality	5	11	.34	Fixed	0.70	(0.47 to 0.92)	<.0001	Random	0.70	(0.47 to 0.92)	<.0001
Lower-limb function score	3	0	.86	Fixed	2.83	(1.99 to 3.67)	<.0001	Random	2.83	(1.99 to 3.67)	<.0001
Bedside balance score	3	89	<.01	Random	0.61	(−0.44 to 1.66)	.25	Fixed	0.49	(0.17 to 0.82)	.003

Pooled estimates were calculated using both fixed-effects and random-effects models. Sensitivity analyses were performed by switching between analytical models.

CI = confidence interval, PTSD = post-traumatic stress disorder.

A funnel plot was constructed for the meta-analysis of anxiety because this outcome included 10 studies (Fig. 10). Visual inspection of the funnel plot suggested possible asymmetry, indicating potential publication bias or small-study effects. However, this finding should be interpreted cautiously because funnel plot asymmetry may arise from factors other than publication bias, including between-study heterogeneity. Therefore, the pooled finding suggesting that VR interventions reduce anxiety in ICU patients should be interpreted with caution. Owing to the limited number of studies available for most outcomes and the considerable clinical and methodological variability across studies, including differences in VR intervention frequency and outcome assessment tools, further subgroup analyses could not be performed.

**Figure 10.**
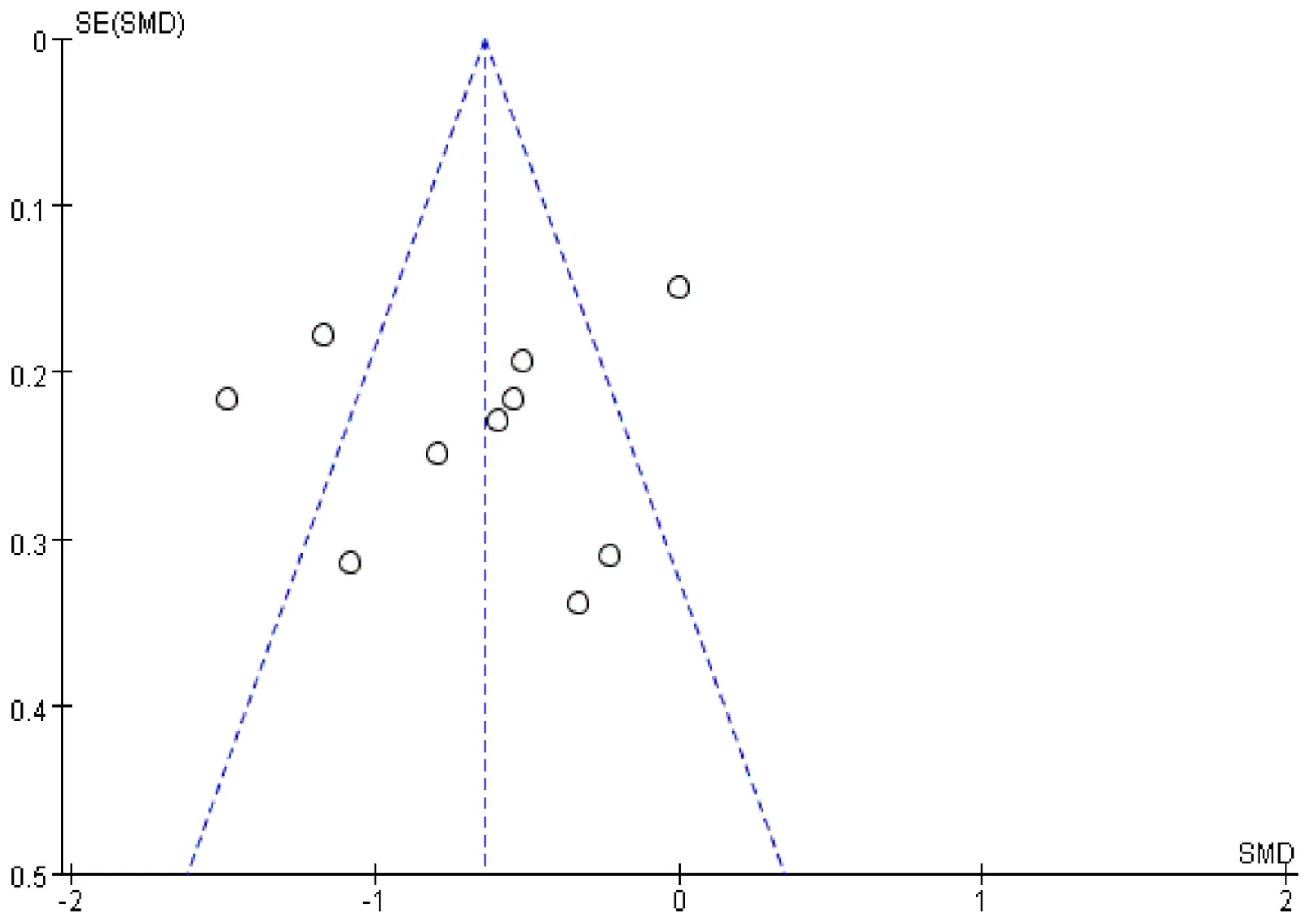
Funnel plot for publication bias of anxiety level. Each dot represents an individual study. SMD = standardized mean difference.

### 3.7. Grading of evidence for meta-analysis results

The certainty of clinical evidence in this meta-analysis ranged from very low to moderate (shown in Table 4). Low-certainty evidence implies limited confidence in the findings, and future research may alter these conclusions. Very-low-certainty evidence suggests that true effects may differ substantially from current estimates, underscoring the need for high-quality studies.^[63]^ Ratings of evidence certainty by the 2 independent reviewers were highly concordant.

**Table 4:** GRADE evidence profile in the meta-analysis of VR.

Items	Certainly assessment							No of patients	Quality
Outcomes	No of studies	Design	Risk of bias	Inconsistency	Indirectness	Imprecision	Other considerations	Total	
Anxiety score	10	Randomized	Serious*	Serious**	Not serious	Not serious	None	913	⊕⊕○○ LOW
Depression score	4	Randomized	Serious*	Serious**	Not serious	Serious***	None	243	⊕⊕○○ Very LOW
Score of PTSD	3	Randomized	Serious*	Not serious	Not serious	Serious‡	None	173	⊕⊕○○ LOW
Pain score	5	Randomized	Serious*	Serious**	Not serious	Not serious	None	504	⊕⊕○○ LOW
Sleep quality	5	Randomized	Not serious	No serious**	Not serious	Serious***	None	328	⊕⊕⊕○ MODERATE
Lower-limb function score	3	Randomized	Serious*	Not serious	Serious†	Serious***	None	192	⊕○○○ VERY LOW
Bedside balance	3	Randomized	Not serious	Serious**	Not serious	Serious***	None	160	⊕⊕○○ LOW

The GRADE approach was used to assess the certainty of evidence, with 4 grades of evidence: high, moderate, low, and very low.

PTSD = post-traumatic stress disorder, VR = virtual reality.

* Lack of allocation concealment and blinding.

** *I^2^* > 75%.

*** Sample size < 400.

† Study populations vary widely.

‡ Wide confidence interval, clinical significance uncertain.

## 4. Discussion

### 4.1. Main findings and comparison with previous reviews

This systematic review synthesized findings from 20 RCTs evaluating the effects of VR interventions on psychological, pain-related, and functional outcomes in adult ICU patients. Overall, VR demonstrated clinically meaningful improvements in anxiety, depression, and PTSD symptoms, and showed potential to enhance sleep quality. Evidence for pain reduction and bedside balance remained inconsistent across studies; no eligible data were available to perform a meta‑analysis of delirium. Notably, in contrast to prior reviews, this analysis detected statistically significant improvements in lower‑limb function, indicating VR could act as a valuable adjunct to early mobilization for specific ICU patients. Substantial heterogeneity, frequently exceeding 80%, is to be anticipated given the inherent characteristics of VR research within this review. Studies differed widely in technological modality (immersive vs semi-immersive), content format (natural scenes, meditation, cognitive tasks, and rehabilitation), intervention duration, session frequency, ICU case-mix, and outcome measurement tools. The inability to perform meaningful subgroup analyses is therefore a recognized limitation and is consistent with previous VR meta-analyses,^[42,43,64]^ which have similarly noted inadequate reporting of critical intervention parameters.

Importantly, this heterogeneity was not consistent across endpoints. Heterogeneity varied markedly by outcome: substantial heterogeneity was found for anxiety (*I^2^* = 81%), pain score (*I^2^* = 96%), and bedside balance (*I^2^* = 89%), whereas depression, PTSD, sleep quality and lower-limb function showed low-to-zero heterogeneity. Sensitivity analyses comparing random-effects and fixed-effects models showed that inter-study heterogeneity could alter the magnitude and statistical significance of pooled effect estimates. For pain, the random-effects model yielded a nonsignificant summary effect (*P* = .23), while the fixed-effects model suggested a statistically beneficial intervention effect. Similarly, pooled results for bedside balance shifted from nonsignificance under the random-effects model (*P* = .25) to statistical significance in the fixed-effects model (*P* = .003). Such model-dependent results reflect large inter-study variability and introduce interpretive uncertainty for these endpoints, partly explaining their low-to-very-low GRADE certainty ratings. Incomplete reporting of key intervention- and patient-level covariates in included trials prevented further investigation into sources of between-study heterogeneity. In contrast, low-to-zero heterogeneity for depression, PTSD, sleep quality and lower-limb function may stem partly from relatively homogeneous intervention targets and outcome assessments across contributing trials.

Despite these methodological constraints, the present review extends previous meta-analyses,^[42,43,64]^ by incorporating updated evidence from 20 RCTs, conducting sensitivity analyses, and evaluating functional outcomes, including lower-limb function and bedside balance. The inclusion of these functional outcomes provides additional evidence regarding the potential rehabilitative effects of VR in ICU patients. However, the small number of studies contributing to the functional analyses and the substantial heterogeneity observed for bedside balance limit the strength of conclusions regarding functional recovery. In addition, inconsistencies in pain outcomes, clinical heterogeneity, small sample sizes, brief intervention durations, and methodological variability further limit the certainty of the overall findings.

### 4.2. Interpretation of psychological outcomes

Across 20 RCTs, the most consistent benefits were observed in anxiety, depression, and PTSD. These findings align with emerging evidence supporting the anxiolytic role of immersive VR in the ICU environment.^[42,43]^ The ICU is inherently distressing, characterized by unfamiliar stimuli, invasive procedures, sleep disruption, and restricted mobility, which contribute to psychological dysregulation.^[39,54]^ VR appears to mitigate these stressors through immersive distraction, affective engagement, and sensory modulation. Many interventions employed natural scenes or meditative content, which are known to activate parasympathetic pathways and counter sympathetic hyperarousal.^[65,66]^

Nevertheless, these psychological outcomes were predominantly assessed using subjective self-report tools. Because VR exposure cannot be concealed, blinding was universally absent across trials, rendering performance bias likely. Patients aware of receiving a “therapeutic” intervention may overreport improvements due to positive expectancy effects.^[67,68]^ Moreover, the high heterogeneity observed across anxiety (*I^2^* > 80%) likely reflects variations in VR content, session duration, baseline psychological distress, and timing of outcome measurement. These methodological inconsistencies introduce uncertainty, supporting the low-to-very-low GRADE ratings for mental health outcomes.

### 4.3. Potential mechanisms of sleep and psychological improvement

The pooled findings suggest potential improvement in sleep quality. While statistical heterogeneity for sleep quality was low (*I^2^* = 11%), a broad spectrum of sleep‑assessment instruments were utilized across included trials. Mechanistically, VR may improve sleep via parasympathetic activation, hypothalamic–pituitary–adrenal‑axis modulation, cortisol reduction, and melatonin regulation.^[46,69,70]^ VR’s immersion may reduce environmental awareness of ICU noise, light, and nocturnal interruptions-common contributors to ICU sleep fragmentation. Yet, the inconsistency of results across trials, limited follow-up, and variability in VR programme design emphasize the need for objective sleep measures such as actigraphy or polysomnography in future research.

Several mechanisms may explain the observed improvements in psychological and sleep outcomes. VR’s immersive environments promote attentional diversion, flow states, and sensory engagement, reducing cognitive load associated with fear, uncertainty, or procedural stress.^[71,72]^ Natural VR scenes activate parasympathetic pathways and attenuate physiological arousal.^[73]^ Meditation-based VR further enhances relaxation and sleep quality by reducing sleep latency, nocturnal awakenings, and sympathetic activity.^[46,74]^ Neuroscientific evidence suggests VR engagement modulates the hypothalamic–pituitary–adrenal axis, lowers cortisol levels, stabilizes circadian rhythms, and enhances melatonin secretion.^[75]^ These overlapping mechanisms support VR as a promising therapeutic modality, although the methodological limitations identified here indicate a need for more rigorous mechanistic investigations.

### 4.4. Pain and motor outcomes

Despite theoretical support for VR in analgesia, the pooled evidence did not demonstrate a statistically significant reduction in pain scores. Several individual studies reported improvements,^[30,39,57]^ but effects were inconsistent and sensitive to modeling assumptions in the sensitivity analyses. The lack of a significant pooled effect likely reflects differences in ICU pain types (procedural, inflammatory, and neuropathic), dosing of VR sessions, and the degree of cognitive engagement. VR may be more effective for affective-emotional pain modulation, but have limited influence over nociceptive or physiological pain.^[76,77]^ Moreover, sedation levels, analgesic use, age, chronic pain history, and emotional distress vary widely across ICU cohorts,^[78]^ influencing responsiveness to VR. These factors, compounded by high heterogeneity, explain the low evidence certainty and the need for targeted VR analgesic protocols.

In contrast to earlier interpretations, the present analysis identified a significant improvement in lower-limb function among VR-exposed patients, with negligible heterogeneity. Many VR interventions incorporated interactive motor tasks, guided movement, or exercise-based programs.^[31,32,58,59]^ This likely reflects VR’s capacity to transform repetitive exercises into engaging, quantifiable tasks, thereby enhancing patient adherence and training dosage (repetitions/duration). These benefits may explain the observed short-term improvements in lower limb function scales.^[79]^ However, bedside balance outcomes did not show additional improvement. The nonsignificant balance effects likely stem from a mismatch between ICU–VR’s seated/supine training modalities (necessitated by hemodynamic instability and medical devices) and the standing weight-transfer demands of true balance rehabilitation.^[80]^ While effectively targeting isolated joint function, current protocols cannot replicate the multisensory challenges (visual-vestibular-proprioceptive conflict and reactive postural adjustments) inherent to balance recovery. Beyond the mismatch of training modes, bedside balance outcomes were associated with substantial heterogeneity (*I^2^* = 89%). This may stem from variable baseline balance capacity among ICU participants, divergent VR rehabilitation protocols (non‑immersive interactive games vs immersive movement tasks), and disparate balance assessment instruments across trials. Sensitivity analyses showed that model selection exerted a prominent influence on pooled balance estimates, which cautioned against overinterpreting this finding.

### 4.5. Implementation considerations

Implementing VR in ICU environments poses practical challenges. ICU patients vary in alertness, respiratory support, sedation level, and physical stability, all of which influence feasibility.^[71,81]^ Immersive VR may produce side effects such as dizziness, vertigo, nausea, or headache.^[56,57]^ Infection control, device sanitization, technical support, and staff training remain essential considerations.^[82]^ Family acceptance, patient willingness, and cultural factors influence uptake and adherence.^[83]^ VR serves as a delivery platform rather than a standalone therapy, with efficacy determined by content quality, interactivity, and patient-specific adaptation. Optimizing outcomes requires tailored VR protocols incorporating targeted cognitive, motor, analgesic, or relaxation modules for distinct ICU populations.^[84]^

### 4.6. Limitations

This review has several limitations. The inclusion of only English and Chinese literature may have omitted recent data from other regions. Significant heterogeneity in intervention protocols, dosages, follow-up durations, and outcome measures reduced comparability and precluded formal subgroup analyses. Reliance on patient-reported outcomes for pain, psychological status, and sleep increased potential bias. Most studies lacked blinding and used usual care as controls, potentially inflating observed benefits. GRADE assessments indicated low or very low certainty for most outcomes, underscoring the need for high-quality, multicenter RCTs with standardized protocols and objective measures. Although some outcomes showed substantial heterogeneity (*I^2^* > 80%), insufficient data precluded robust subgroup analyses or meta-regression. Future studies should standardize VR protocols and reporting to facilitate such analyses.

## 5. Conclusion

This systematic review and meta-analysis provide updated and comprehensive evidence on the effects of VR interventions in adult ICU populations. VR demonstrates consistent benefits in reducing anxiety, depression, and PTSD symptoms, and shows promising effects on sleep quality. In contrast, evidence for analgesia and bedside balance remain inconclusive, while improvements in lower-limb function suggest potential value as an adjunct to early mobilization in selected patients. However, substantial heterogeneity, methodological limitations, small sample sizes, lack of blinding, and predominance of subjective outcome measures resulted in low or very low certainty of evidence for outcomes. These findings indicate that VR may serve as a supportive, nonpharmacological intervention to promote psychological well-being and sleep in conscious adult ICU patients, although its role in pain control, cognitive outcomes, and functional recovery requires further clarification. To meaningfully advance the field, future studies should adopt standardized VR protocols, incorporate objective physiological and functional measures, recruit adequately powered samples, and explore population- and condition-specific VR applications. Robust multicenter trials integrating patient, family, and clinician perspectives will be essential to establish the safety, feasibility, and clinical effectiveness of VR within the complex ICU environment.

## Author contributions

**Conceptualization:** Xihui Sun, Vimala Ramoo, Nor’azim Mohd Yunos.

**Data curation:** Xihui Sun, Jiangping Ma.

**Formal analysis:** Xihui Sun, Jiangping Ma.

**Methodology:** Vimala Ramoo, Jiangping Ma.

**Project administration:** Xihui Sun.

**Supervision:** Vimala Ramoo, Nor’azim Mohd Yunos.

**Writing – original draft:** Xihui Sun.

**Writing – review & editing:** Xihui Sun, Vimala Ramoo.




