# Supplementary material for: The overall effects of virtual reality interventions on ICU patients: A systematic review and meta-analysis
Source: Medicine (Baltimore). 2026 Oct 23;105(43):e51180. doi: 10.1097/MD.0000000000051180 (PMC13706371; doi:10.1097/MD.0000000000051180)
Supplement: Supplementary file 1 [file medi-105-e51180-s001.doc]

Supplementary material1 :

Search strategy-I**（taking PubMed as an example）**

#1 "Virtual Reality"[Mesh] OR "virtual reality"[tiab] OR "VR"[tiab] OR "VR technology"[tiab] OR "VR devices"[tiab] OR "immersive virtual reality"[tiab] OR "virtual reality therapy"[tiab] OR "electronic relaxation devices"[tiab]

#2 "Pain"[Mesh] OR "pain management"[Mesh] OR "Analgesia"[Mesh] OR "pain"[tiab] OR "pain relief"[tiab] OR "analgesia"[tiab] OR "pain intensity"[tiab] OR "anxiety"[tiab] OR "post-traumatic stress disorder"[tiab] OR "post-intensive care syndrome"[tiab] OR "mental health"[tiab] OR "motor function"[tiab] OR "sleep quality"[tiab]

#3 "Intensive Care Units"[Mesh] OR "Critical Care"[Mesh] OR "ICU"[tiab] OR "critical care"[tiab] OR "critically ill patients"[tiab] OR "intensive care patients"[tiab] OR "ventilated patients"[tiab]

#4 "Randomized Controlled Trial"[pt] OR "Controlled Clinical Trial"[pt] OR "randomised controlled trials"[tiab] OR "clinical randomised trials"[tiab] OR "quasi-experimental"[tiab] OR "feasibility study"[tiab] OR "pilot study"[tiab]

#5 #1 AND #2 AND #3 AND #4

#6 "Animals"[Mesh] NOT "Humans"[Mesh]

#7 #5 NOT #6

Search strategy-II**（taking CNKI as an example）:**

(SU=('虚拟现实' + 'VR' + 'VR技术' + 'VR设备' + '沉浸式虚拟现实' + '虚拟现实疗法' + '电子放松设备') OR TI=('虚拟现实' + 'VR'))

AND

(SU=('疼痛' + '疼痛管理' + '镇痛' + '疼痛缓解' + '焦虑' + '创伤后应激障碍' + '重症监护后综合征' + '心理健康' + '运动功能' + '睡眠质量') OR FT=('疼痛评分' + '疼痛强度'))

AND

(SU=('重症监护' + 'ICU' + '危重患者' + '重症患者' + '机械通气患者') OR TI=('重症监护室'))

AND

(SU=('随机对照试验' + '临床随机试验' + '准实验' + '类实验' + '可行性研究') OR FT=('试点研究'))

NOT SU=('动物' + '小鼠' + '大鼠' + '基础研究')
