# Supplementary material for: The overall effects of virtual reality interventions on ICU patients: A systematic review and meta-analysis
Source: Medicine (Baltimore). 2026 Oct 23;105(43):e51180. doi: 10.1097/MD.0000000000051180 (PMC13706371; doi:10.1097/MD.0000000000051180)
Supplement: Supplementary file 2 [file medi-105-e51180-s002.doc]

**S2-1: Cochrane's assessment items for RCT studies**

1. Method of randomization of cases.
2. Concealment of allocation.
3. Blinding of patients and investigators.
4. Blinding of outcome assessors.
5. Completeness of data.
6. Whether selective reporting.
7. Whether there is other bias

Refer to: <https://www.cochrane.org/.>
